# Associations between change in labour market policies and work stressors: a comparative longitudinal survey data analysis from 27 European countries

**DOI:** 10.1186/s12889-020-09364-3

**Published:** 2020-09-10

**Authors:** T. Lunau, M. Wahrendorf, N. Dragano, J. Siegrist, K. A. van der Wel, M. Rigó

**Affiliations:** 1 Institute of Medical Sociology, Centre for Health and Society, Medical Faculty of the University of Düsseldorf, Düsseldorf, Germany; 2 Senior professorship on work stress research, Medical Faculty, University of Düsseldorf, Düsseldorf, Germany; 3 Department of Social Work, Child Welfare and Social Policy, Oslo Metropolitan University, Oslo, Norway

**Keywords:** Work stressors, Effort-reward imbalance, Job strain, Cross-national study, Labour market policies

## Abstract

**Background:**

Many studies have shown that work stressors have a negative impact on health. It is therefore important to gain an understanding of how work stressors can be reduced. Recent studies have shown that employees in countries with high investments into labour market policies less often report exposure to work stressors. Although these studies are indicative of an influence of the political level on work stressors, they are based on cross-sectional cross-country analyses where causal assumptions are problematic. The aim of this study is to extend the existing evidence by longitudinally testing whether changes in labour market policies are related to changes in work stressors.

**Methods:**

We used comparative longitudinal survey data from the European Working Conditions Survey (27 countries; for the years 2005, 2010, 2015). The measurement of work stressors is based on two established work stress models: effort-reward imbalance (ERI) and job demand-control (job strain). To measure labour market policies, we used information on active (ALMP) and passive labour market policies (PLMP). After excluding persons with missing data, 64,659 participants were eligible for the ERI and 67,114 for job strain analyses. Estimation results are provided by three-way multilevel models (individuals, country-years, country), which allow us to estimate longitudinal and cross-country macro-effects.

**Results:**

An increase in ALMP leads to a decrease of ERI. The analyses for the subcomponents ‘effort’ and ‘reward’ showed that mainly the ‘reward’ component is positively associated with ALMP. The association between ALMP and ‘reward’ shows that an increase in ALMP investments is related to an increase in rewards. Yet, no significant longitudinal associations between ALMP and job strain, and between PLMP and the work stressors, were observed.

**Conclusions:**

The study extends the current knowledge with longitudinal information by showing that an increase in ALMP is associated with an increase in rewards and a decrease of ERI. These longitudinal analyses may support a causal interpretation. The findings of this study have important policy implications. Our main result suggests that investments into ALMP can lead to better working conditions.

## Background

Psychosocial work stressors have a negative impact on health as they can lead to stress-related diseases such as depression [1, 2], cardiovascular diseases [3], musculoskeletal diseases [4] or diabetes [5]. To protect workers’ health, it is important to understand how these work stressors can be reduced. Evidence based health promotion in the workplace usually focuses on the individual himself [6] or on the organisational level of the company [7] in order to reduce or prevent stressful work, or to optimise coping processes of the individual person. In addition to the individual and organisational level, working conditions are also influenced by structural factors (e.g. economy, digitalisation). One such factor concerns national labour policies [8]. The explicit aim of these policies is to regulate the labour market, improve working conditions and provide adequate social protection in critical situations, e.g. in case of job loss [9]. Labour policies can be further divided into protective and integrative policies [10, 11]. Protective policies aim to provide support in critical life situations. Passive labour market policies (PLMP) are an example of protective policies. PLMP refer to public expenditures aiming to compensate individuals, e.g. in case of wage loss in a period of unemployment. In countries with a high level of PLMP, individuals and families can maintain a normal and socially acceptable standard of living regardless of their market performance. Integrative labour policies, in contrast, are designed to integrate disadvantaged people into working life. Active labour market policies (ALMP) can usually be categorised as integrative labour policies. These policies aim at preventing unemployment and promoting employment [12]. Examples of ALMP expenditures are vocational training programs, which can lead to a better match between job seekers and the job vacancies [9]. The possibility to expand one’s own skills and knowledge over the course of working life is important in order to cope with the changing demands of the labour market (for example digitalisation processes).

Theoretically, there are several possible mechanisms involved in how ALMP and PLMP can impact psychosocial working conditions. First, generous PLMPs allocate bargaining power to employees and labour unions by lifting the reservation wage [13]. For instance, in countries with high investments into PLMP it should be easier to quit a job with unfavourable working conditions (e.g. effort-reward imbalance, job strain) compared to countries with low investments. In countries with low investments into PLMP the individual depends strongly on gainful employment and the person may even be forced to accept stressful working conditions [14]. This may improve working conditions directly, through negotiations between employees, their unions and employers. Second, in countries with high investments into PLMP unemployed people can invest more time into their job search improving subsequent job match and job quality [15–17]. Therefore, in this case PLMP would indirectly lead to better working conditions. Third, PLMP also includes investments into early retirement and this could directly affect psychosocial working conditions in the case of partial early retirement. Older people who work full-time and experience stressful working conditions could benefit from a reduced working time and therefore working conditions are directly improved. Furthermore, the opportunity for older people to enter a full early retirement could indirectly lead to a lower work stress level in a country. If older people with problems in the labour market and higher stress levels have the opportunity to leave the labour market, it is possible that the work stress level in a country decreases because these individuals experiencing high stress levels are out of the labour market (healthy worker effect). Fourth, in countries where unemployed workers are trained and receive the opportunity to acquire new skills through investment into ALMP, there should also be a better match between skills and job requirements [16]. We assume that this will indirectly lead to lower psychosocial work stress levels in a country as higher levels of job control and reward at work can be expected [18].

Previous research provided initial findings that ALMP and PLMP are related to psychosocial work stressors [8, 10, 18]. These studies have found that in countries with high investments into labour market policies, psychosocial work stressors were less often reported than in countries with low investments into such policies. However, there are still important research gaps as the number of studies is small and the studies suffer from methodological problems. Most of the cross-country studies, for instance, are restricted to a limited number of countries. Consequently, we are less able to generalise these findings. Furthermore, the results of multilevel models may often be biased due to the insufficient number of countries included in the analysis, which again limits the opportunity to control for country differences.

Even more important is the problem that causality was difficult to establish in these studies, mainly because the time perspective has rarely been considered in the cross-sectional studies conducted so far. This makes it difficult to draw a clear conclusion in the sense that the implementation or increase of policy measures leads to an improvement of working conditions. Therefore, longitudinal studies looking at policy changes in conjunction with changes of work stressors at the individual level are needed. Approaches taking into account both the cross-country and temporal dimensions are especially promising as they combine the advantages of both types of research [19].

With this study, we extend current knowledge by focusing on both between-country variation and within-country changes, taking advantage of the opportunity for longitudinal modelling provided by the repeated surveys in the European Working Conditions Survey (EWCS). We carry out a comparative analysis with 27 European countries surveyed in three consecutive waves of a cross-national survey over a 10-year period.

## Methods

### Data

Data were obtained from the European Working Conditions Survey (EWCS) [20]. The EWCS is a periodical survey conducted by Eurofound with a repeated cross-sectional design. It started in 1990 with ongoing waves every five years. Available data from the EWCS include individual information on working conditions for many European countries at different points in time. This kind of dataset is also referred to as comparative longitudinal survey data [19]. The dataset includes non-repeated observations at the individual level and repeated observations at the population level from 27 European countries, thus offering the opportunity to compare both between and within higher-level units. Therefore, we can measure differences between countries and changes within countries over time. Data on psychosocial work stressors are available in the most recent three waves of the EWCS (years 2005, 2010 and 2015), providing information from 27 European countries (*n* = 95,739). The country sample sizes are around 1000 in each wave with a few exceptions where around 2000 employees are interviewed. The aim of the EWCS is to draw random samples in each country that are representative of those aged 15 and over who are in employment. To achieve this, in each country, a multistage, stratified random sampling method was used to recruit a sample from the working population aged ≥15 years. Details on the survey are provided elsewhere [21–23]. The response rate varies between countries and years, with the lowest response rate in Sweden in the year 2015 (11%) and the highest response rate in Cyprus in the year 2015 (69%). For the analyses, we exclude employed individuals above the age of 65 (2603), because they may have particular work situations (e.g. work after pension age). For similar reasons, we also exclude persons working < 8 h per week (1368) and those who were self-employed (12,935). After excluding persons with missing data on outcome and covariates, a total of 64,659 participants were eligible for the analysis of ERI as a work stressor and 67,114 participants for the analysis of job strain as a work stressor.

### Measures

#### Psychosocial work stressors

The measurement of the work stressors are based on established work stress models, which have previously been linked to health outcomes. We used the available survey items of the EWCS to measure ERI based on the effort-reward imbalance [24] and job strain based on the demand-control model [25]. The list of the underlying survey items can be found in Additional file 1.

Effort-reward imbalance is defined as the ratio of effort and reward. ‘Effort’ is measured with four items (e.g. job involves working at very high speed – Cronbach’s alpha 0.64). Occupational rewards were measured with five items (e.g. financial reward – Cronbach’s alpha 0.50).

Job strain is defined as the ratio of demand and control. Higher values are indicating higher job strain. Demand is defined identically to effort. The construct of ‘control’ consists of two sub-dimensions: skill discretion and decision authority. The control dimension was measured with 10 items (5 items skill discretion and 5 items decision authority; Cronbach’s alpha 0.70).

The number of response categories vary between the items used to define effort, reward and control. We followed previous studies using the EWCS [26, 27] and standardized the values of the response categories (see Additional file 1). As such, all single items and the composite constructs of effort, control and reward have a range between 1 and 2, with higher values indicating higher levels of effort, reward or control. To build the scales for effort, reward and control we used the mean value of the sum of the respective items.

Furthermore, we constructed two summary indices, an effort-reward imbalance [28, 29] scale and a scale measuring job strain (the combination of effort and control) [25, 30, 31]. To do this, we divided the effort scale by the reward/control scale, following a proposed procedure to operationalise these two models [29]. The range of these two scales varies between 0.5 and 2.0. Higher values indicate higher levels of ERI or job strain and therefore higher work stress.

Although the original questionnaires to measure the effort-reward imbalance and the demand-control model were not included in the EWCS, the questionnaire offers a number of adequate items to operationalise the two models. To conduct sensitivity analyses we repeated the computations for a longer period (5 waves; years 1995 to 2015) where we used a shorter measure of the effort and control scales due to restricted data availability for the longer period (results not shown).

#### Policy indicators

We use two policy indicators of national labour market policies (LMP). As described in the introduction, these indicators cover two relevant dimensions of labour market policies, i.e., ‘integrative’ and ‘protective’ labour market policies. In the case of protective policies, we use an indicator that summarises the amount of a country’s labour market expenditures on ‘passive labour market policies’ (PLMP), expressed as a percentage of GDP. To measure integrative policies, we use an indicator measuring the amount of investments into ALMP. Information is again expressed as a percentage of GDP and comprises various policy measures of ALMP (for a detailed description of the policy indicators see Additional file 2. For data sources, see Table 1). Thus, our LMP measures, instead of showing the absolute value of spending by country, capture the priority of labour market policies in the countries’ own political agenda. The unemployment rate is not included as a separate item in the analyses. Instead, we followed previous literature and used the unemployment rate to adjust the LMP measures for need [32]. To do this, we used the ratio between ALMP (or PLMP) and unemployment rate. This prevents the possibility that a country’s higher expenditures were simply related to higher levels of unemployment. The unemployment rate for each country is also shown in Table 1. We used the information from the years 2005, 2010 and 2015. We also included GDP (GDP per capita in current US$ available from the World Bank) as a macro-level control variable into our models. All macro level indicators are presented in Table 1.

**Table 1:** Macrolevel indicators for 2005, 2010 and 2015

Country	ALMP^a^			PLMP^b^			GDP per capita^c^			Unemployment rate^d^		
	2005	2010	2015	2005	2010	2015	2005	2010	2015	2005	2010	2015
Belgium	0.47	0.52	0.52	2.28	2.21	1.71	36,967.26	44,380.18	40,431.95	8.50	8.30	8.50
Bulgaria	0.40	0.09	0.14	0.20	0.43	0.40	3869.53	6843.27	6993.78	10.10	10.30	9.20
Czech Republic	0.11	0.21	0.30	0.22	0.35	0.19	13,346.18	19,808.07	17,715.62	7.90	7.30	5.10
Denmark	1.22	1.63	1.65	2.28	1.73	1.27	48,799.82	58,041.41	53,254.85	4.80	7.50	6.20
Germany	0.81	0.52	0.27	1.92	1.28	0.88	34,696.62	41,785.56	41,394.66	11.20	7.00	4.60
Estonia	0.05	0.13	0.10	0.12	0.85	0.42	10,338.31	14,638.60	17,412.45	8.00	16.70	6.20
Greece	0.06	0.22	0.21	0.40	0.71	0.48	22,551.74	26,917.76	18,167.77	10.00	12.70	24.90
Spain	0.63	0.75	0.45	1.43	3.05	1.98	26,510.72	30,736.63	25,817.39	9.20	19.90	22.10
France	0.66	0.75	0.66	2.01	1.94	2.04	34,760.19	40,638.33	36,613.38	8.90	9.30	10.40
Ireland	0.50	0.73	0.48	0.79	2.78	1.22	50,878.64	48,711.95	61,908.79	4.60	14.60	10.00
Italy	0.46	0.32	0.41	0.67	1.32	1.29	31,959.26	35,849.37	30,170.52	7.70	8.40	11.90
Cyprus	–	0.25	0.12	–	0.62	0.80	24,959.27	30,818.48	23,217.48	5.30	6.30	15.00
Latvia	0.15	0.52	0.10	0.30	0.70	0.41	7558.74	11,326.22	13,639.69	10.00	19.50	9.90
Lithuania	0.14	0.22	0.25	0.12	0.47	0.22	7863.16	11,984.87	14,291.91	8.30	17.80	9.10
Luxembourg	0.45	0.49	0.60	0.66	0.79	0.69	80,289.70	104,965.31	100,428.37	4.60	4.60	6.50
Hungary	0.23	0.54	0.82	0.38	0.71	0.24	11,205.97	13,092.23	12,503.68	7.20	11.20	6.80
Malta	–	0.05	0.10	–	0.33	0.20	15,835.35	21,087.79	23,715.53	6.90	6.80	5.40
Netherlands	0.80	0.73	0.51	1.73	1.43	1.79	41,577.16	50,950.03	45,175.23	5.90	5.00	6.90
Austria	0.44	0.64	0.57	1.45	1.36	1.47	38,403.13	46,858.04	44,176.67	5.60	4.80	5.70
Poland	0.35	0.59	0.38	0.85	0.34	0.27	8021.00	12,597.86	12,556.36	17.90	9.70	7.50
Portugal	0.49	0.54	0.48	1.24	1.44	1.36	18,784.95	22,538.65	19,252.63	8.80	12.00	12.60
Romania	0.11	0.03	0.02	0.39	0.54	0.11	4676.32	8209.92	8977.50	7.10	7.00	6.80
Slovenia	0.19	0.39	0.16	0.40	0.69	0.52	18,169.18	23,437.47	20,873.16	6.50	7.30	9.00
Slovak Republic	0.16	0.23	0.16	0.26	0.59	0.33	11,669.42	16,600.61	16,182.30	16.40	14.50	11.50
Finland	0.70	0.83	0.85	1.82	1.71	1.93	38,969.17	46,202.42	42,494.66	8.40	8.40	9.40
Sweden	0.89	0.84	1.01	1.22	0.76	0.55	43,085.35	52,132.92	50,832.55	7.70	8.60	7.40
United Kingdom	0.04	0.07	–	0.17	0.28	–	41,732.64	39,079.84	44,472.15	4.80	7.80	5.30
Mean	0.42	0.48	0.44	0.93	1.09	0.88	26,943.66	32,601.25	31,210.04	8.23	10.12	9.40

^a^Expenditure on active labour market policies in percentage of GDP (Source: OECD Public expenditure and participant stocks on LMP dataset https://stats.oecd.org/. In order to also include Bulgaria, Cyprus, Malta and Romania in the analyses, the OECD data were supplemented by information from the European Commissions LMP:EXPSUMM dataset https://webgate.ec.europa.eu/empl/redisstat/databrowser/view/LMP_EXPSUMM/default/table?category=lmp_expend)

^b^Expenditure on passive labour market policies in percentage of GDP (Source: OECD Public expenditure and participant stocks on LMP dataset https://stats.oecd.org/)

^c^GDP in current US-Dollar (Source: Worldbank https://data.worldbank.org/indicator/NY.GDP.PCAP.CD)

^d^ Unemployment in percent of active population (Source: Eurostat une_rt_a dataset https://appsso.eurostat.ec.europa.eu/nui/show.do?dataset=une_rt_a&lang=en). The unemployment rate is used to adjust the LMP measures for need. To do this, we used the ratio between LMP measures and unemployment rate

Additionally, we included age (<=30, 30–55, 55–64), gender (male, female), contract (permanent contract, fixed term contract, temporary employment, apprenticeship or other), branches (NACE categories) and occupational position into the models in order to control for compositional differences, that is, the fact that European populations have a different composition in terms of several individual characteristics. The measurement of occupational position is based on the International Standard Classification of Occupations (ISCO) developed by the International Labour Office [33]. The ISCO classifies jobs into 390 different categories. For our analyses, we used the broader hierarchical structure of the ISCO, which is based on four different skill levels: Skill level 4 – high skilled clerical (e.g. managers); Skill level 3 – low skilled clerical (e.g. secretaries); Skill level 2 – high skilled manual (e.g. mechanical engineers); Skill level 1 – low skilled manual (e.g. kitchen helpers). The skill level refers to the complexity of the tasks in the current job of the interviewed person. A sample description is presented in Table 2.

**Table 2:** Sample description (data are presented as mean (SD) for continuous measures, and n (%) for categorical measures)

	Sample using ERI		Sample using Job Strain	
	***N*** = 64,659		***N*** = 67,114	
ERI	.85	(.18)	–	–
Job Strain	–	–	.90	(.21)
Effort	1.39	(.22)	1.39	(.22)
Reward	1.65	(.18)	–	–
Control	–	–	1.57	(.23)
Age	41.26	(11.36)	41.43	(11.41)
Survey wave				
2005 - 4th EWCS	17,540	(27.1%)	17,089	(25.5%)
2010 - 5th EWCS	24,654	(38.1%)	25,727	(38.3%)
2015 - 6th EWCS	22,465	(34.7%)	24,298	(36.2%)
Gender				
Male	30,485	(47.1%)	31,562	(47.0%)
Female	34,174	(52.9%)	35,552	(53.0%)
ISCO skill level				
ISCO1	13,108	(20.3%)	13,893	(20.7%)
ISCO2	8483	(13.1%)	8818	(13.1%)
ISCO3	28,928	(44.7%)	29,970	(44.7%)
ISCO4	14,140	(21.9%)	14,433	(21.5%)
Employment contract				
A permanent contract	51,873	(80.2%)	53,513	(79.7%)
Fixed term contract	7454	(11.5%)	7731	(11.5%)
Temporary employment agency contract	909	(1.4%)	970	(1.4%)
Apprenticeship or other training scheme	425	(0.7%)	443	(0.7%)
Other	3998	(6.2%)	4457	(6.6%)
NACE				
Agriculture, hunting, forestry and fishing	1401	(2.2%)	1463	(2.2%)
Industry	15,555	(24.1%)	16,132	(24.0%)
Services	24,333	(37.6%)	25,341	(37.8%)
Public administration and defence; compulsory social sec	4967	(7.7%)	5088	(7.6%)
Other services	18,403	(28.5%)	19,090	(28.4%)

### Statistical analyses

**Fig. 1:**
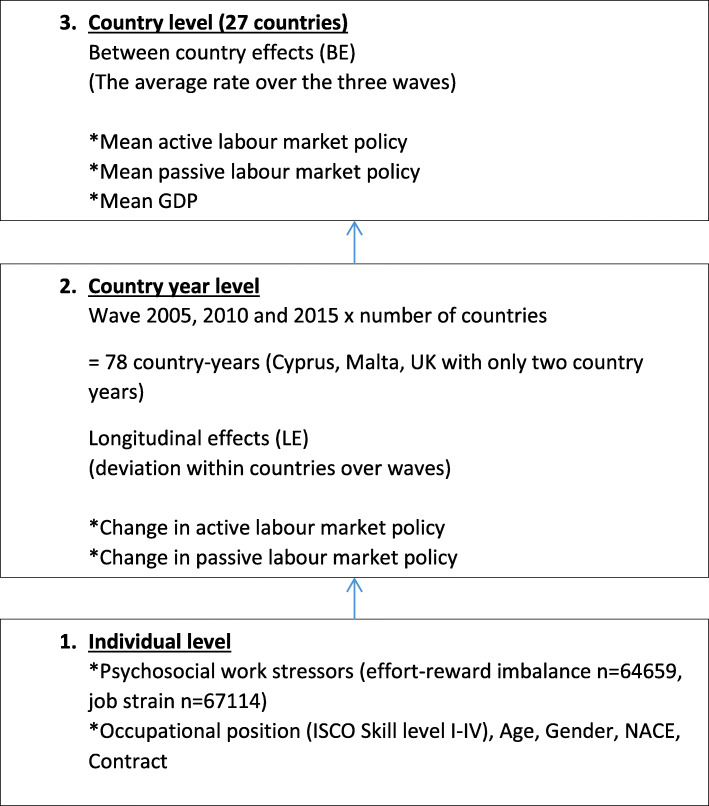
Presentation of the three-level multilevel model

To carry out our analysis we use multilevel linear models relying on the hierarchical structure of the EWCS: Individuals (level 1) are clustered in country-years (level 2), which means that countries are observed in several consecutive waves. These country-years are then again clustered in countries (level 3). The data structure is also displayed in Fig. 1. Such datasets are also called as comparative longitudinal survey data including longitudinal information on higher-group units (in our case, countries) over time [19]. The full information for the measurement of ERI and job strain is available in three waves of the EWCS (years 2005, 2010, 2015) for 27 European countries. Taking advantage of the unique structure of the dataset, we are able to analyse the role of country characteristics that are constant (time invariant characteristics on the country level – level 3) and country characteristics that change over time (time varying characteristics on the country year level – level 2). Specifically, we can test if the level of psychosocial work stressors is lower in countries with a higher average spending of LMP, and we can test if an increase in the expenditure on LMP within countries is related to a decrease in psychosocial work stressors. This technique allows us to simultaneously analyse between country effects (BE) and longitudinal effects (LE) [19, 34]. To do this, the macro variables (ALMP, PLMP and GDP) are decomposed into two parts: a variable that measures the between-country variation, and a variable measuring the within-country variation. Specifically, for the between-country variation, the models include the mean of the macro-level variable across all years for each country (capturing enduring cross-national differences). Variations over time, in turn, are analysed by including the differences to the country mean for each country-year (capturing changes within countries over time). As a result, the original macro-level variables appear in a cross-sectional (country-level mean across years; e.g. mean value of ALMP over the years 2005, 2010 and 2015) and in a longitudinal dimension (deviation from the country mean; e.g. deviation of the annual spending of ALMP from the mean value). The longitudinal dimension gives us the opportunity to directly test if a change in LMP is related to a change in psychosocial work stressors. This is not possible with data only using cross-country information. Furthermore, by construction, this longitudinal estimate is not biased by unobserved country-level time-invariant confounders and is identical to an estimate produced by a fixed-effect panel regression [35]. Failing to control for time-invariant unobservable confounders (e.g. country-specific cultural attitudes, social norms, traditions, which may be correlated with both work stressors and macro policies) can be a major problem when observational data are used to test causal assumptions.

We also include a dummy variable for time, to control for the possibility of simultaneous but unrelated time trends in the dependent and independent variables. We use six different models to analyse the associations between the macro-level determinants and the two work stress models and their subcomponents. The first model (model 0), called ‘empty or zero model’ presents the variance composition for the work stressors. In the next model (model 1) individual covariates are included. These first two models are presented in Table 3*.* The models 2–5 include the macro-level indicators, and the respective results are presented in Table 4 to Table 6. The accepted level of significance is *p* ≤ 0.05. We additionally conducted sensitivity analyses and used macro-level variables measured one, two and three years before each EWCS wave (lagged effects). With these analyses, we can test if working conditions are not primarily linked to expenditure in the same single year, but to the prevailing level of spending in the years prior to the observation year. All calculations are based on Stata 15.

**Table 3:** Association between individual variables and work stressors (based on linear multilevel models)

	ERI		Effort		Reward		Job Strain		Control	
	b	p	b	p	b	p	b	p	b	p
**Model 0**										
Variance										
Level 3 (Country)	.0007		.0032		0.0019		.0017		.0046	
Level 2 (Country-years)	.0004		.0004		0.0008		.0004		.0005	
Level 1 (Individual)	.0326		.0449		0.0312		.0418		.0479	
**Model 1**										
**Level 1** *(Individuals)*										
Year (ref. 2005)										
2010	−.0001	.992	−.0113	.072	−.0131	.039	.0007	.914	−.0156	.013
2015	−.0107	.055	−.0030	.938	.0215	.001	.0064	.313	−.0157	.013
Age (ref. <=30)										
30 < age < 55	.0063	≤.001	−.0096	≤.001	−.0242	≤.001	−.0193	≤.001	.0229	≤.001
age > =55	−.0087	≤.001	−.0447	≤.001	−.0378	≤.001	−.0433	≤.001	.0233	≤.001
Gender (ref. male)										
female	.0199	≤.001	.0090	≤.001	−.0244	≤.001	.0338	≤.001	−.0461	≤.001
ISCO (ref. ISCO1)										
ISCO2	−.0020	.445	.0319	≤.001	.0357	≤.001	−.0253	≤.001	.0681	≤.001
ISCO3	−.0275	≤.001	.0163	≤.001	.0638	≤.001	−.0741	≤.001	.1328	≤.001
ISCO4	−.0406	≤.001	.0353	≤.001	.1100	≤.001	−.1248	≤.001	.2445	≤.001
Contract (ref. permanent contract)										
fixed term contract	.0318	≤.001	−.0120	≤.001	−.0652	≤.001	.0215	≤.001	−.0404	≤.001
temporary employment	.0615	≤.001	−.0064	.364	−.1082	≤.001	.0625	≤.001	−.0914	≤.001
apprenticeship	−.0357	≤.001	−.0373	≤.001	.0230	.010	−.0214	.024	−.0232	.017
other	.0170	≤.001	−.0295	≤.001	−.0595	≤.001	−.0118	≤.001	−.0111	.001
NACE (ref. agriculture)										
industry	.0393	≤.001	.0633	≤.001	.0007	.880	.0543	≤.001	−.0209	≤.001
services	.0296	≤.001	.0409	≤.001	−.0076	.112	.0365	≤.001	−.0229	≤.001
public administration	−.0116	.036	.0123	.058	.0328	≤.001	−.0144	.016	.0262	≤.001
other services	−.0043	.402	−.0022	.719	.0021	.662	−.0126	.023	.0032	.509
Constant	.7996	≤.001	1.3890	≤.001	1.7494	≤.001	.8127	≤.001	1.7326	≤.001
Variance										
Level 3 (Country)	.0007		.0035		.0017		.0017		.0032	
Level 2 (Country-years)	.0003		.0004		.0005		.0004		.0004	
Level 1 (Individual)	.0317		.0439		.0289		.0385		.0404	
N	64,659		64,659		64,659	67,114		67,114		

Model 0 presents the variance of the outcome variable on the individual (level 1), the country-year (level 2) and the country level (level 3)

**Table 4:** Association between macro level variables and work stressors (ERI, Job Strain; based on linear multilevel models)

	ERI								Job Strain							
	Model 2		Model 3		Model 4		Model 5		Model 2		Model 3		Model 4		Model 5	
	b	p	b	p	b	p	b	p	b	p	b	p	b	p	b	p
**Level 2 (Country-years)**																
ALMP (LE)	−.3691	(.005)	−.3613	(.007)					−.0702	(.658)	−.0459	(.775)				
PLMP (LE)					−.0657	(.411)	−.0679	(.396)					−.00037	(.968)	−.0074	(.935)
GDP (LE)			−.0003	(.699)			−.0007	(.430)			−.0010	(.326)			−.0010	(.286)
**Level 3 (Country)**																
ALMP (BE)	.1401	(.210)	.0706	(.625)					−.0722	(.663)	−.2705	(.189)				
PLMP (BE)					.0880	(.176)	.0513	(.548)					.0235	(.808)	−.0550	(.662)
GDP (BE)			.0003	(.442)			.0002	(.510)			.0008	(.123)			.0005	(.334)
Constant	.7920	(≤.001)	.7860	(≤.001)	.7901	(≤.001)	.7844	(≤.001)	.8168	(≤.001)	.7996	(≤.001)	.8101	(≤.001)	.7991	(≤.001)
Variance																
Level 3 (Country)	.0007		.0008		.0007		.0007		.0017		.0016		.0017		.0017	
Level 2 (Country-years)	.0003		.0003		.0003		.0003		.0004		.0004		.0005		.0004	
Level 1 (Individual)	.0317		.0317		.0317		.0317		.0385		.0385		.0385		.0385	
N	64,659		64,659		64,659		64,659		67,114		67,114		67,114		67,114	

All models are adjusted for the following level 1 individual characteristics: year, age, gender, ISCO, contract and NACE. BE (between effects) refers to the country mean of the respective macro variable over the three waves, LE (longitudinal effects) refers to the within-country variation of the macro variable

## Results

Table 3 presents the results of the empty model (Model 0) and the multilevel models with individual control variables (Model 1). Model 0 presents the variance composition for the work stressors. Albeit the variance is largest at the individual level, there still is variation between countries, as well as between country-years (e.g. for ERI the country level explains 2% of the total variation (Var Lev 3 / Var Lev 3 + Var Lev 2 + Var Lev 1), country-year level explains 1% and the individual level explains 97%). The coefficients for the variable ‘year of data collection’ in model 1 indicate the trend of the work stressors between the years 2005 and 2015 (reference 2005). Thereby, while the pattern is not clear for the two work stressors ERI and job strain, we find an increase for the component ‘reward’, and a decrease for the component ‘control’ between the years 2005 and 2015. Model 1 also presents the associations between individual variables and the different work stressors. These latter results show (1) that women have higher levels of work stressors than men, (2) that lower-skilled occupational groups have higher levels of work stressors compared to higher-skilled occupational groups, and (3) that employees with fixed-term contracts or temporary employment relationships report higher work stressors than employees with a permanent contract. After including the individual variables into the model, a small decrease for the individual variance is observed. Table 4 displays the results for models 2–5, where the macro-level indicators (LMP and GDP) are additionally included. As described in the methods section, we decomposed the macro variables into the ‘between-country’ (country mean of the respective macro variables, BE) and the ‘within-country’ components (within country variation of the macro variables over time, LE). Table 4 shows a significant longitudinal effect between ALMP and ERI. An increase in ALMP investments is related to a decrease of the work stressor ERI. No association between ALMP and ERI is obvious based on cross-sectional between-country comparison. When looking at the subcomponents of effort and reward, the model shows that an increase of ALMP over time (LE) is related to an increase of reward within countries (Table 5). The between-country association (BE) between ALMP items and ‘reward’ indicates that countries with higher ALMP measures also have higher reward scores. After controlling for GDP, the between-country differences are no longer significant. We did not find significant associations between the ALMP indicators (both BE and LE) and job strain (Table 4). For the sub-dimension of ‘control’, results show significant effects between ALMP(BE)/PLMP(BE) and control (Table 6). However, there is no significant longitudinal association (LE) between change over time in ALMP or PLMP and control. The results from the additional analyses using lagged effects do not differ significantly from the results presented here.

**Table 5:** Association between macro level variables and work stressors (Effort, Reward; based on linear multilevel models)

	Effort								Reward							
	Model 2		Model 3		Model 4		Model 5		Model 2		Model 3		Model 4		Model 5	
	b	p	b	p	b	p	b	p	b	p	b	p	b	p	b	p
**Level 2 (Country-years)**																
ALMP (LE)	−.1763	(.268)	−.1624	(.316)					.4427	(.003)	.4367	(.004)				
PLMP (LE)					.0163	(.859)	.0143	(.877)					.1219	(.182)	.1267	(.168)
GDP (LE)			−.0006	(.571)			−.0007	(.478)			.0002	(.813)			.0007	(.477)
**Level 3 (Country)**																
ALMP (BE)	.5487	(.008)	.2408	(.334)					.3547	(.018)	.1184	(.508)				
PLMP (BE)					.3359	(.004)	.1671	(.255)					.2179	(.0113)	.0812	(.439)
GDP (BE)			.0013	(.048)			.0012	(.066)			.0010	(.034)			.0009	(.040)
Constant	1.359	(≤.001)	1.3349	(≤.001)	1.3500	(≤.001)	1.3311	(≤.001)	1.7293	(≤.001)	1.7134	(≤.001)	1.7232	(≤.001)	1.7121	(≤.001)
Variance																
Level 3 (Country)	.0028		.0024		.0027		.0024		.0014		.0012		.0013		.0011	
Level 2 (Country-years)	.0004		.0004		.0005		.0005		.0004		.0004		.0005		.0005	
Level 1 (Individual)	.0439		.0439		.0439		.0439		.0289		.0289		.0289		.0289	
N	64,659		64,659		64,659		64,659		64,659		64,659		64,659		64,659	

All models are adjusted for the following level 1 individual characteristics: year, age, gender, ISCO, contract and NACE. BE (between effects) refers to the country mean of the respective macro variable over the three waves, LE (longitudinal effects) refers to the within-country variation of the macro variable

**Table 6:** Association between macro level variables and work stressors (Control; based on multilevel models)

	Control							
	Model 2		Model 3		Model 4		Model 5	
	b	p	b	p	b	p	b	p
**Level 2 (Country-years)**								
ALMP (LE)	−.0544	(.734)	−.0764	(.638)				
PLMP (LE)					.0252	(.783)	.0291	(.751)
GDP (LE)			.0009	(.379)			.0009	(.385)
**Level 3 (Country)**								
ALMP (BE)	.7398	(≤.001)	.7206	(.001)				
PLMP (BE)					.3502	(.002)	.2895	(.048)
GDP (BE)			.0001	(.888)			.0004	(.509)
Constant	1.6914	(≤.001)	1.6931	(≤.001)	1.6923	(≤.001)	1.6893	(≤.001)
Variance								
Level 3 (Country)	.0018		.0019		.0023		.0024	
Level 2 (Country-years)	.0005		.0005		.0005		.0005	
Level 1 (Individual)	.0404		.0404		.0404		.0404	
N	67,114		67,114		67,114		67,114	

All models are adjusted for the following level 1 individual characteristics: year, age, gender, ISCO, contract and NACE. BE (between effects) refers to the country mean of the respective macro variable over the three waves, LE (longitudinal effects) refers to the within-country variation of the macro variabl

## Discussion

This contribution studied the associations between national-level labour policy indicators and work stress. We used data from the EWCS covering 27 countries surveyed in the years 2005, 2010 and 2015. These so-called comparative longitudinal survey data gave us the opportunity to test, for the first time, longitudinal relationships between national labour policies and individual work stressors.

The major drawback of previous studies [10, 36, 37] on the association between labour market policies and work stressors is their reliance on cross-country comparisons, which limits their relevance to policy interventions. In short, cross-sectional data do not tell us much about change. Hence, the adequacy of cross-sectional analyses for studying the question of whether LMP will lead to better working conditions is questionable. In contrast, this study uses longitudinal data at the country level to address the relation between changes in national policies and changes in the level of work stress. A further problem with observational studies in general is that unmeasured confounding may bias the results. In this study, it is possible to control for time-constant unobserved confounders at the country level (e.g. work culture in countries) by separating the macro variables into two terms: one term capturing changes of work stressors within countries, and another one disentangling differences between countries.

The results of this study partly support our hypothesis that an increase in labour market policy investment results in a decrease of work stressors. Specifically, our results demonstrated a statistically significant association between an increase in ALMP investments and a decrease of ERI. The analyses for the subcomponents effort and reward revealed that this result is driven by ‘reward’, suggesting that an increase in ALMP investments leads to an increase in rewards. Concerning the complementary work stress model, no significant associations were observed in the longitudinal dimension between ALMP and job strain. The same holds true for associations between PLMP and the two work stress models under study.

Furthermore, it was possible to test the cross-country associations between ALMP and work stressors in 27 European countries. The results of the ‘between-country’ comparisons partly support former findings. Previously, it has been shown that countries with higher spending into ALMP and PLMP have, on average, lower levels of work stress [10, 36]. In this study, we found that employees in countries with higher spending in ALMP and PLMP report higher levels of reward and control, compared to employees from countries with lower investments. In contrast to the former studies, we also included GDP into our analyses to control for the economic situation of the countries. After the inclusion of GDP, the association between ALMP and reward lost its statistical significance. This observation points to the fact that it is important to control for other potential confounding factors at the macro level when cross-country comparisons are analysed. However, as GDP and ALMP are highly correlated, respective macro-level effects cannot be disentangled. It is therefore not justified to conclude that no association between labour market policies and reward has been documented. While previous studies found significant associations between labour market policies and ERI [36, 37], implying that higher levels of spending are related to lower levels of ERI, the results from this study could not replicate this finding. There may be several reasons for this discrepancy. For instance, the operationalisation of ERI differs between studies. In addition, in former studies fewer countries were included in the analyses. It is possible that the country composition influenced the results.

Although we assumed that levels of psychosocial work stressors are lower in countries with higher average spending on LMP (BE) and that an increase in LMP spending (LE) is related to a decrease of work stressors, we found inconsistencies between the BE and LE estimates for some work stressors. We found a significant LE but no significant BE for ALMP and ERI / Reward in our specification including GDP. One possible explanation for this apparent inconsistency might be the high correlation between levels of GDP and ALMP. For instance, the specification without GDP (Model 2) indicates that reward is, on average, higher in countries spending a higher fraction of their GDP on ALMP (BE estimate); and reward will get higher in countries if they decide to increase to spend a higher fraction of their GDP on ALMP (LE estimate). However, the BE estimate loses its significance in the specification including GDP (Model 3), suggesting that the between-country differences in reward might be driven by GDP. Nonetheless, the cross-country association between ALMP and reward cannot be ruled out because of the high correlation between GDP and the ALMP measure. It should be also noted that the significant LE estimate in both Model 2 and 3 accentuate the policy implication of our result: the within-country longitudinal impact on reward holds regardless of a country’s GDP. Another possible explanation for this inconsistency might be a lack of measurement equivalence of psychosocial work stressors between countries. For PLMP and control we found a significant BE but no LE. In this case, one possible explanation is that the significant between country association is not causal. It is possible that other country specific influences that are related to PLMP and control act as a confounder and therefore we didn’t find a significant LE of PLMP and control.

The results obtained in the present study extend the current knowledge on the determinants of work stressors, giving first indications that an increase in ALMP investments may improve stress-reducing occupational rewards. Although this is the first study that tests the longitudinal relationship between change in labour market investments and change in work stressors directly, several studies analysed the relationship between specific working conditions and LMP investments, relying exclusively on cross-country comparison (see above [10, 36]). Additionally, a large body of research in economics evaluates specific ALMPs in terms of various labour market outcomes, such as re-employment or wages [12, 38]. These studies usually rely on data from experimental randomised control trials or on large-scale individual-level observations. Results suggest that ALMPs have the potential to increase the employability of unemployed people in the medium and longer run [14, 35], improve subsequent earnings and job stability [15]. This latter impact is explained by a higher job match. Our results are in line with the findings of the ALMP evaluation literature as job security and income satisfaction are part of the reward measure. The inconsistency of our findings regarding the two constructs of ‘control’ and ‘reward’ may be due to the fact that ALMP measures are more in line with strengthening reward than control.

Research on non-labour market impacts of ALMPs, such as health or well-being, is scarce. The current paper contributes to filling this gap in the literature by analysing the impact of ALMPs on work stressors. The available previous results point to positive impacts on health and quality of life [39–41]. Relying on the association between health outcomes and work stressors [1–5], the results of the current paper support the notion that a reduction of work stress (ERI) and an increase of rewards is partly due to an increase in ALMP investments.

This study has several limitations. First, the EWCS does not include the original, psychometrically validated questionnaires to measure effort-reward imbalance [29] and job strain [30]. Therefore, we used the available items on psychosocial working conditions and developed proxy measures to operationalise both work stress models. This procedure has also been applied in former studies with EWCS data [26]. We have also tested the internal consistency of the scales of effort, reward and control (Cronbach’s alpha) and the criterion validity of ERI and job strain by using logistic regression models to test the associations between these two stressors and self-reported health. The results show significant associations between ERI / job strain and poor self-reported health. The Cronbach’s alpha values lie between 0.50 and 0.70 and are lower than the internal consistency found for the original instruments [29]. Therefore, a stronger case would have been provided if the full original questionnaires were applied. Second, our analyses on the longitudinal dimension is based on only three time points. Although the time span for the analyses is 10 years, more waves would have provided a more robust test of the longitudinal associations. However, it was possible to apply a shortened version of the effort and the control dimensions for the analysis of a longer time period (5 waves; 1995, 2000, 2005, 2010, 2015). We have also carried out the analyses for effort, control and the job strain scale covering this longer time period, but we didn’t observe a substantial change in the results. As a third limitation, although the EWCS comprise a higher number of countries than was the case in former studies, there still might be a bias of the results due to unobserved heterogeneity. The low number of level 3 units (countries) prevents an appropriate inclusion of control variables at this level. Therefore, confounding problems can occur, especially so in the ‘between-country’ analyses. As described in more detail above, the longitudinal analyses allow to control for time-invariant variables. Therefore, they are better suited to test the causal assumption that labour market policy measures lead to better working conditions in the society. Nevertheless, it is possible that unobserved time-variant country-level characteristics that are associated with LMP and psychosocial work stressors act as confounders. A change of government leading to modifications in LMP but also to adjustments in other political areas that are related to working conditions (e.g. occupational safety and health regulations) is one example. Deindustrialisation may also confound the association by replacing unhealthy routine jobs with more knowledge-intensive jobs, which again forces governments to invest more heavily in ALMP. While much of this will be captured by the time-trends in our statistical models, we cannot exclude the possibility that countries are affected differently by this process. Fourth, the response rates vary between time and countries and are rather low in some cases. This may also have biased the results. However, in further analyses we have received no indication that the results are biased (correlation between the response rate and the psychosocial work stressors; analyses were adjusted for response rate; Sweden was excluded from the analyses due to a low response rate). Finally, the indicators of labour market policies are expenditure-based and it is unclear if they also reflect the specific quality of these policy measures [42]. Countries vary regarding the composition of ALMP spending, design, implementation, target group and participation rate. All these factors influence the effectiveness of the programs [38, 39]. As such, we cannot analyse which program attributes contribute most to lowering work stress. However, this kind of detailed internationally comparable dataset is currently not available. Fifth, the operationalisation of LMP as a percentage of GDP has implications for interpretation as LMP changes do not necessarily reflect real changes in absolute terms. For instance, if GDP increases and LMP remains stable in terms of absolute values, LMP investment as a percentage of GDP will shrink. In this paper, however, we wanted to shed light on changes in ‘LMP policy effort’ which gives us an indication of the priority of labour market policies in a country. Future research should also address whether our results hold for absolute changes in LMP investment too.

Despite these limitations the study has several strengths. To our knowledge, it is the first time that the association between national policy measures and work stressors have been analysed with comparative longitudinal survey data. The dataset includes information from 27 countries from 3 waves covering a period of 10 years. With this kind of dataset it is possible to test structural between-country variations and longitudinal within-country effects. Previous studies were based on cross-sectional between-country comparisons and included fewer countries. The longitudinal dimension offers an opportunity to test the causal assumption that an increase in labour market policies leads to better working conditions. Although the original questionnaires to measure established stress models are not available in the EWCS, the various psychosocial work stressors that are measured in the EWCS allow us to build proxy measures for two central work stressors: effort-reward imbalance and job strain. Moreover, we used well-established indicators of labour market policies which are comparable between countries.

## Conclusions

This study specifies and extends the current knowledge on the influence of country-specific labour market policies and work stressors. It complements former cross-national studies by showing that mainly the resource components of the work stress models are associated with national labour policies. Countries with higher investments into ALMP and PLMP show higher rewards and higher control compared to countries with lower investments. The study extends the current knowledge with longitudinal information by showing that an increase in ALMP is associated with an increase in rewards and with a decrease of ERI. The main result thus suggests that investments into ALMP can improve psychosocial working conditions.

## Supplementary information

**Additional file 1 :** List of underlying EWCS survey items.

**Additional file 2 :** Policy indicators.

## Data Availability

The EWCS datasets are stored with the UK Data Service (UKDS) in Essex, UK and are publicly available via their website (https://ukdataservice.ac.uk/). Users are required to be registered with the UK Data Service. Users who register have to accept the End User Licence (EUL) which is agreed to during the registration process. The data of the macrolevel indicators are available via the website of OECD (https://stats.oecd.org/.), the European Commission (https://webgate.ec.europa.eu/empl/redisstat/databrowser/view/LMP_EXPSUMM/default/table?category=lmp_expend), Eurostat (https://appsso.eurostat.ec.europa.eu/nui/show.do?dataset=une_rt_a&lang=en) and the Worldbank (https://data.worldbank.org/indicator/NY.GDP.PCAP.CD).

## Abbreviations

ALMP: Active labour market policies
BE: Between country effects
ERI: Effort-reward imbalance
EWCS: European working conditions survey
ISCO: International standard classification of occupations
LE: Longitudinal effects
LMP: Labour market policies
NACE: Nomenclature statistique des activités économiques dans la Communauté européenne
PLMP: Passive labour market policies
