# Correction to: Associations between change in labour market policies and work stressors: a comparative longitudinal survey data analysis from 27 European countries

**DOI:** 10.1186/s12889-020-09647-9

**Published:** 2020-10-19

**Authors:** T. Lunau, M. Wahrendorf, N. Dragano, J. Siegrist, K. A. van der Wel, M. Rigó

**Affiliations:** 1 Institute of Medical Sociology, Centre for Health and Society, Medical Faculty of the University of Düsseldorf, Düsseldorf, Germany; 2 Senior professorship on work stress research, Medical Faculty, University of Düsseldorf, Düsseldorf, Germany; 3 Department of Social Work, Child Welfare and Social Policy, Oslo Metropolitan University, Oslo, Norway

**Correction to: BMC Public Health 20, 1377 (2020)**

**https://doi.org/10.1186/s12889-020-09364-3 **

It was highlighted that in the original article [1] there was a misalignment in Tables 3 and 5. This Correction article shows the correct Tables 3 and 5. The original article has been updated.

**Table 3:** Association between individual variables and work stressors (based on linear multilevel models)

		ERI		Effort		Reward		Job strain		Control	
		b	p	b	p	b	p	b	p	b	p
**Model 0**											
Variance	Level 3 (Country)	.0007		.0032		0.0019		.0017		.0046	
	Level 2 (Country-years)	.0004		.0004		0.0008		.0004		.0005	
	Level 1 (Individual)	.0326		.0449		0.0312		.0418		.0479	
**Model 1**											
**Level 1** *(Individuals)*											
Year (ref. 2005)	2010	−.0001	.992	−.0113	.072	−.0131	.039	.0007	.914	−.0156	.013
	2015	−.0107	.055	−.0030	.938	.0215	.001	.0064	.313	−.0157	.013
Age (ref. <=30)	30 < age < 55	.0063	≤.001	−.0096	≤.001	−.0242	≤.001	−.0193	≤.001	.0229	≤.001
	age > =55	−.0087	≤.001	−.0447	≤.001	−.0378	≤.001	−.0433	≤.001	.0233	≤.001
Gender (ref. male)	female	.0199	≤.001	.0090	≤.001	−.0244	≤.001	.0338	≤.001	−.0461	≤.001
ISCO (ref. ISCO1)	ISCO2	−.0020	.445	.0319	≤.001	.0357	≤.001	−.0253	≤.001	.0681	≤.001
	ISCO3	−.0275	≤.001	.0163	≤.001	.0638	≤.001	−.0741	≤.001	.1328	≤.001
	ISCO4	−.0406	≤.001	.0353	≤.001	.1100	≤.001	−.1248	≤.001	.2445	≤.001
Contract (ref. permanent contract)	fixed term contract	.0318	≤.001	−.0120	≤.001	−.0652	≤.001	.0215	≤.001	−.0404	≤.001
	temporary employment	.0615	≤.001	−.0064	.364	−.1082	≤.001	.0625	≤.001	−.0914	≤.001
	apprenticeship	−.0357	≤.001	−.0373	≤.001	.0230	.010	−.0214	.024	−.0232	.017
	other	.0170	≤.001	−.0295	≤.001	−.0595	≤.001	−.0118	≤.001	−.0111	.001
NACE (ref. agriculture)	industry	.0393	≤.001	.0633	≤.001	.0007	.880	.0543	≤.001	−.0209	≤.001
	services	.0296	≤.001	.0409	≤.001	−.0076	.112	.0365	≤.001	−.0229	≤.001
	public administration	−.0116	.036	.0123	.058	.0328	≤.001	−.0144	.016	.0262	≤.001
	other services	−.0043	.402	−.0022	.719	.0021	.662	−.0126	.023	.0032	.509
Constant		.7996	≤.001	1.3890	≤.001	1.7494	≤.001	.8127	≤.001	1.7326	≤.001
Variance	Level 3 (Country)	.0007		.0035		.0017		.0017		.0032	
	Level 2 (Country years)	.0003		.0004		.0005		.0004		.0004	
	Level 1 (Individual)	.0317		.0439		.0289		.0385		.0404	
N		64,659		64,659		64,659		67,114		67,114	

Note: Model 0 presents the variance of the outcome variable on the individual (level 1), the country year (level 2) and the country level (level 3)

**Table 5:** Association between macro level variables and work stressors (Effort, Reward; based on linear multilevel models)

		Effort								Reward							
		Model 2		Model 3		Model 4		Model 5		Model 2		Model 3		Model 4		Model 5	
		b	p	b	p	b	p	b	p	b	p	b	p	b	p	b	p
**Level 2**	ALMP (LE)	−.1763	(.268)	−.1624	(.316)					.4427	(.003)	.4367	(.004)				
*(Country Year)*	PLMP (LE)					.0163	(.859)	.0143	(.877)					.1219	(.182)	.1267	(.168)
	GDP (LE)			−.0006	(.571)			−.0007	(.478)			.0002	(.813)			.0007	(.477)
**Level 3**	ALMP (BE)	.5487	(.008)	.2408	(.334)					.3547	(.018)	.1184	(.508)				
*(Country)*	PLMP (BE)					.3359	(.004)	.1671	(.255)					.2179	(.0113)	.0812	(.439)
	GDP (BE)			.0013	(.048)			.0012	(.066)			.0010	(.034)			.0009	(.040)
Constant		1.359	(≤.001)	1.3349	(≤.001)	1.3500	(≤.001)	1.3311	(≤.001)	1.7293	(≤.001)	1.7134	(≤.001)	1.7232	(≤.001)	1.7121	(≤.001)
Variance	Level 3 (Country)	.0028		.0024		.0027		.0024		.0014		.0012		.0013		.0011	
	Level 2 (Country years)	.0004		.0004		.0005		.0005		.0004		.0004		.0005		.0005	
	Level 1 (Individual)	.0439		.0439		.0439		.0439		.0289		.0289		.0289		.0289	
N		64,659		64,659		64,659		64,659		64,659		64,659		64,659		64,659	

Note: All models are adjusted for the following level 1 individual characteristics: year, age, gender, ISCO, contract and NACE. BE (between effects) refers to the country mean of the respective macro variable over the three waves, LE (longitudinal effects) refers to the within-country variation of the macro variable
