# Supplementary material for: Associations between change in labour market policies and work stressors: a comparative longitudinal survey data analysis from 27 European countries
Source: BMC Public Health. 2020 Sep 10;20:1377. doi: 10.1186/s12889-020-09364-3 (PMC7488105; doi:10.1186/s12889-020-09364-3)
Supplement: Supplementary file 1 — Additional file 1 : List of underlying EWCS survey items. [file 12889_2020_9364_MOESM1_ESM.docx]

**Additional file 1. List of underlying EWCS survey items.**

| **Work stress measure** | **EWCS item** | **Response categories** |
| --- | --- | --- |
| **Job strain (Psychological demands/Control)** | |  |
| **Psychological demand** | Does your work include working at a very high speed? | 1 (never) to 7 (all of the time) |
|  | Does your work include working to tight deadlines? | 1 (never) to 7 (all of the time) |
|  | Does your work include interruptions due to unforeseen tasks? | 1 (never) to 4 (very often) |
|  | You have enough time to get the job done | 1 (always) to 5 (never) |
| **Skill discretion** | Does your job include learning new things? | 1 (no) to 2 (yes) |
|  | Does your job include solving complex tasks? | 1 (no) to 2 (yes) |
|  | Does your job include monotonous tasks | 1 (no) to 2 (yes) |
|  | Does your job include repetitive tasks of less than 10 minutes? | 1 (no) to 2 (yes) |
| **Decision authority** | Are you able to choose the order of the tasks? | 1 (no) to 2 (yes) |
|  | Are you able to choose the methods of your work? | 1 (no) to 2 (yes) |
|  | Are you able to choose the rate or speed of your work? | 1 (no) to 2 (yes) |
| **ERI (effort/reward)** |  |  |
| **Effort** | Does your work include working at a very high speed? | 1 (never) to 7 (all of the time) |
|  | Does your work include working to tight deadlines? | 1 (never) to 7 (all of the time) |
|  | Does your work include interruptions due to unforeseen tasks? | 1 (never) to 4 (very often) |
|  | You have enough time to get the job done | 1 (always) to 5 (never) |
| **Reward** | I might loose my job in the next 6 months | 1 (strongly agree) to 5 (strongly disagree) |
|  | My job offers good prospects for career advancement | 1 (strongly disagree) to 5 (strongly agree) |
|  | Your job gives you the feeling of work well-done | 1 (never) to 5 (always) |
|  | Considering all my efforts and achievements in my job, I feel I get paid appropriately | 1 (strongly disagree) to 5 (strongly agree) |
|  | Your colleagues and/or manager support you | 1 (none) to 2 (at least colleagues or manager) |

Survey items were standardized in the following way:

$${item\_st}_{i}=1+\frac{\left( {item\_orig}_{i}-1 \right)}{x-1}$$

where *x* refers to the number of response categories as shown in the table.
