# Supplementary material for: Associations between change in labour market policies and work stressors: a comparative longitudinal survey data analysis from 27 European countries
Source: BMC Public Health. 2020 Sep 10;20:1377. doi: 10.1186/s12889-020-09364-3 (PMC7488105; doi:10.1186/s12889-020-09364-3)
Supplement: Supplementary file 2 — Additional file 2 : Policy indicators. [file 12889_2020_9364_MOESM2_ESM.docx]

Additional file 2 Policy indicators

| **Protective labour market policies** | | |
| --- | --- | --- |
|  | PLMP | Passive labour market policies refer to public expenditures that aim to compensate individuals, both in the case of (1) a loss of wage or salary and (2) of involuntary early retirement. In the analysis, the indicator is measured in percentage of GDP. |
| **Integrative labour market policies** | | |
|  | ALMP | Active labour market policies refer to public expenditures that aim to promote labour market integration for groups that are disadvantaged in the labour market (including unemployed people seeking a new job). It comprises (1) training programs (workplace training or further education) (2) job rotation and sharing (skill enlargement) (3) supported employment and rehabilitation (services for people with limited working capacity) (4) direct job creation (provision of jobs in the public sector) and (5) start-up incentives (grants provided to start-up business). In the analysis, the indicator is measured in percentage of GDP. |
